# Anterior mediastinal tracheostomy for severe tracheal stenosis in a child with severe motor and intellectual disabilities: a case report

**DOI:** 10.1186/s40792-023-01712-w

**Published:** 2023-07-11

**Authors:** Tsuyoshi Iwanaka, Takeshi Shiraishi, Ryuichiro Hirose, Toshihiko Sato

**Affiliations:** Department of General Thoracic, Breast and Pediatric Surgery, Fukuoka University School of Medicine, 7-45-1 Nanakuma, Jonan-Ku, Fukuoka, 814-0180 Japan

**Keywords:** Severe motor and intellectual disabilities, Tracheal stenosis, Tracheoinnominate artery fistula, Tracheostomy, Laryngotracheal separation, Anterior mediastinal tracheostomy, Grillo

## Abstract

**Background:**

Owing to recurrent aspiration pneumonia and airway stenosis secondary to thoracic deformities, tracheostomy or laryngotracheal separation are often necessary in children with severe motor and intellectual disabilities. However, these procedures are associated with the risks of tracheal stenosis due to tracheal granulation and tracheoinnominate artery fistula formation. We report a case of a child with severe motor and intellectual disabilities treated with an anterior mediastinal tracheostomy.

**Case presentation:**

The patient was a 15-year-old boy with severe motor and intellectual disabilities due to intractable epilepsy. Due to thoracic deformity and tracheomalacia, the patient had a flattened and narrowed trachea. Accordingly, laryngotracheal separation was performed 4 months before admission to avoid aspiration pneumonia. Due to a common cold, the patient required frequent sputum suctioning, which exacerbated the tracheal stenosis. Bronchoscopy revealed tracheal stenosis 4–5 cm caudal to the tracheostomy site, tracheal mucosal ulcers, and pulsation of the innominate artery on the anterior wall of the trachea. We performed an anterior mediastinum tracheostomy to release the tracheal stenosis and prevent tracheoinnominate artery fistula formation.

**Conclusions:**

Anterior mediastinal tracheostomy has several advantages. Including sufficient release of bony compression, release of tracheal hyperextension, and relief of tracheal and innominate artery contact ensures a cannula-free tracheostomy, and there is no need to dissect the brachiocephalic artery. It is the procedure of choice in cases of head and neck malignancies requiring extensive tracheal resection and could be a good surgical option for severe tracheal stenosis and tracheoinnominate artery fistula in children with severe motor and intellectual disabilities.

## Background

Children with severe motor and intellectual disabilities (SMID) are more susceptible to aspiration pneumonia due to dysphagia and gastroesophageal reflux caused by progressive changes to the trunk of the body, such as scoliosis secondary to muscle hypertonia. Furthermore, thoracic and spinal deformities can lead to compression of the mediastinal structures and subsequently to airway stenosis induced by compression of the sternum, vertebral body, and the brachiocephalic artery [1–3]. To improve the quality of life of these children and their caretakers, tracheostomy and laryngotracheal separation are commonly performed to prevent severe aspiration pneumonia and gastroesophageal reflux. However, these procedures sometimes increase the likelihood of tracheal stenosis by stretching the tracheal formation or intratracheal granulation caused by tracheal tube contact or frequent suctioning [4]. Among these complications, tracheoinnominate artery fistula (TIF) has a poor prognosis with a survival rate of 14.3% [5] and measures should be implemented to ensure its prompt diagnosis and intervention. TIF is caused by the weakening of the tracheal and brachiocephalic artery walls due to chronic mechanical stimulation from the tracheal cannula. TIF frequency is reported as 0.2–0.7% after tracheostomy [6] and 3–12% after laryngotracheal separation [7–9]. The higher frequency of TIF after laryngotracheal separation can possibly be attributed to the anterior displacement of the trachea, which can then easily be compressed by the innominate artery [8]. We report a case of a child with SMID treated with an anterior mediastinal tracheostomy with tracheal interposition after total manubrial removal to resolve the tracheal stenosis and prevent TIF formation.

## Case presentation

The patient was a 15-year-old boy with SMID due to intractable epilepsy and with secondary tracheomalacia. The patient had undergone laryngotracheal separation 4 months before admission to prevent recurrent aspiration pneumonia. After developing a common cold, the patient required frequent sputum suctioning due to increased mucus production. Wheezing and retractive breathing became progressively prominent, cyanosis appeared, and the patient was transferred to our hospital. Bronchoscopy revealed flattened tracheal stenosis with intratracheal granulation. The patient required tracheal tubing at the stenosis site to stabilize the respiratory status. After the general condition was stabilized, we performed a detailed workup.

**Fig. 1:**
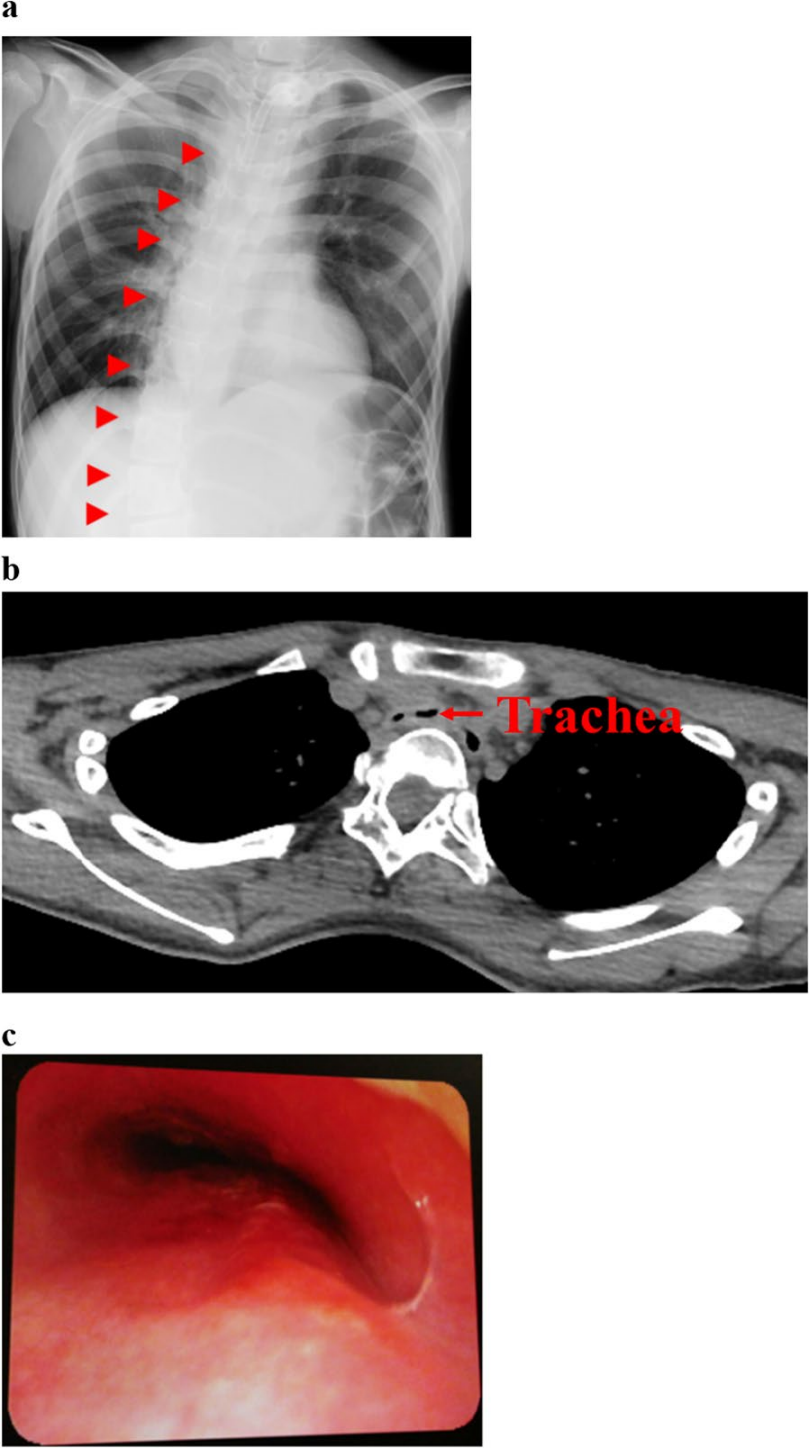
Preoperative findings. **a** Chest radiograph showing scoliosis (red arrowheads). **b** Computed tomography image demonstrates the stenosed trachea between the sternum, vertebral body, and innominate artery. **c** Bronchoscopy reveals a flattened trachea 4–5 cm caudal to the tracheostomy site, tracheal mucosa ulcers, and pulsation of the innominate artery on the anterior wall of the trachea

Chest radiograph showed scoliosis (Fig. 1a), and chest computed tomography (CT) images showed tracheal stenosis between the sternum, vertebral body, and innominate artery (Fig. 1b). Bronchoscopy revealed a flattened trachea 4–5 cm caudal to the tracheostomy site, tracheal mucosal ulcers, and pulsation of the innominate artery on the anterior wall of the trachea (Fig. 1c). Although cannula-free tracheostomy is preferred to prevent the progression of tracheal mucosal ulcers, a tracheostomy tube had to be placed to achieve adequate ventilation.

The following factors particularly complicated the patient condition: (1) the tracheal stenosis was partly caused by the hyperextension of the trachea due to the laryngotracheal separation; (2) the airway compression by the sternum, vertebral body, and innominate artery worsened the tracheal stenosis; (3) the tracheomalacia was exacerbated by edematous changes in the tracheal mucosa due to inflammation caused by the common cold and the frequent sputum suctioning; (4) the continuous placement of the tracheostomy tube caused ulcers of the tracheal mucosa; and (5) the innominate artery pulsation was observed on the anterior tracheal wall and thus the separation of the trachea and innominate artery is necessary. To manage these obstacles, we considered the following approaches: (1) manubrial removal to release tracheal compression by the bony thorax; (2) repositioning of the trachea to the right side of the innominate artery, creating a new permanent tracheal stoma on the anterior chest wall to release overtension and eliminate contact with the innominate artery; and (3) cannula-free management to prevent tracheal granulation. After this comprehensive consideration, we decided to perform an anterior mediastinum tracheostomy with tracheal repositioning.

**Fig. 2:**
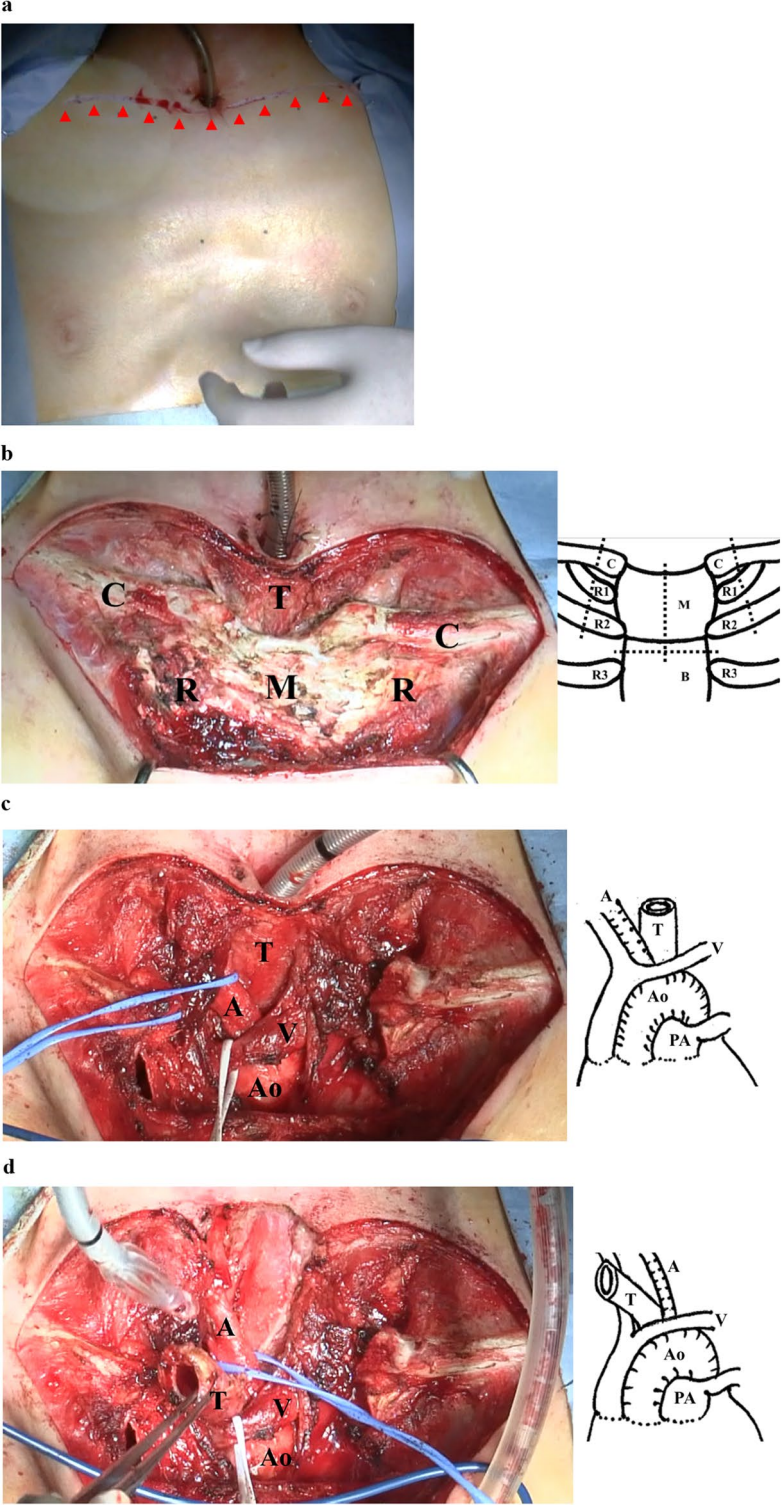

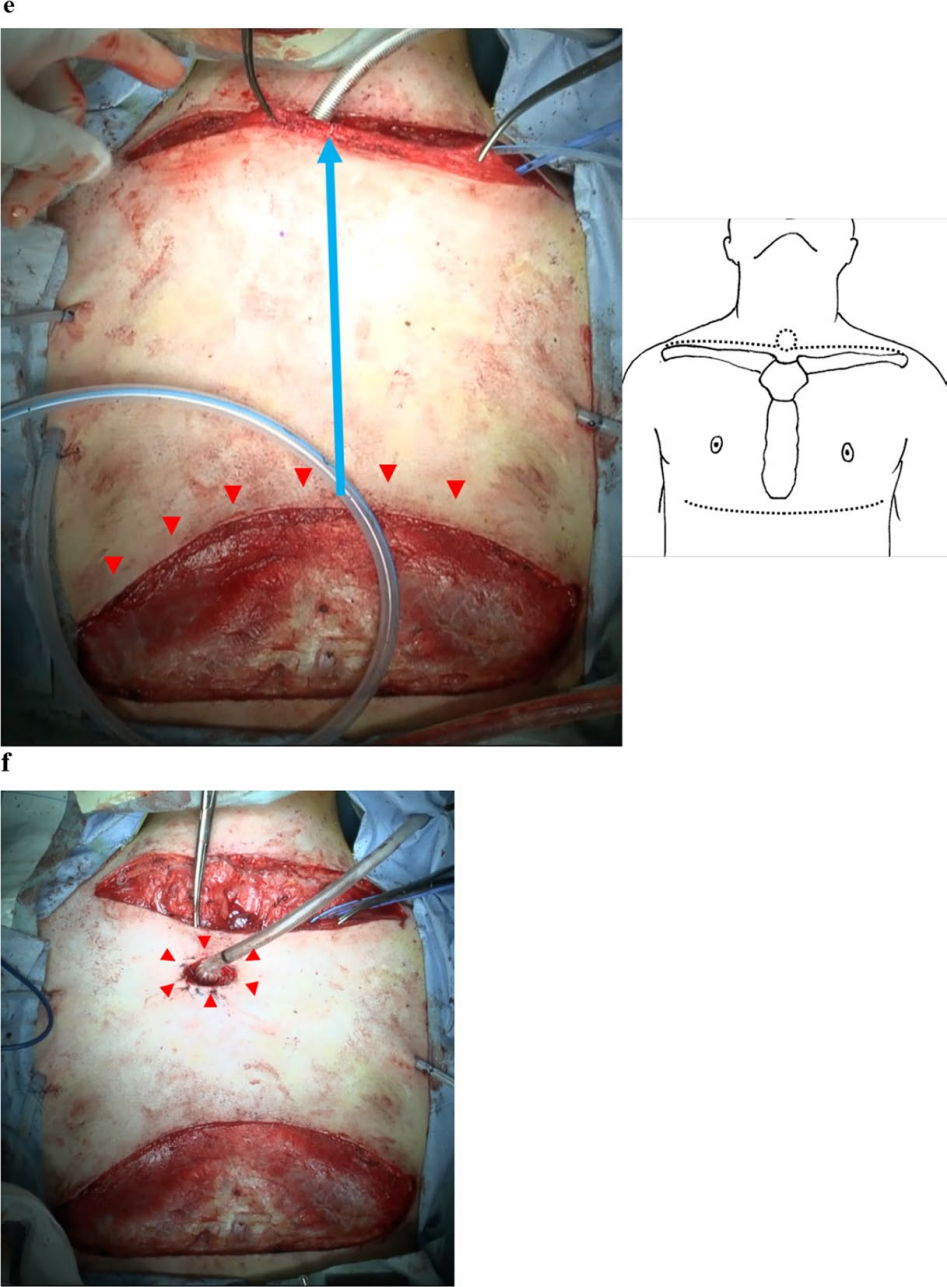
Intraoperative photographs. **a** The initial transverse incision following the clavicular line with the existing tracheal stoma as the center point. **b** Exposing the bony chest wall. Dashed lines indicate the clavicle, sternum, and rib dissection lines. **c** The bony chest wall is resected. **d** The trachea is repositioned to the right side of the ascending aorta and innominate artery. **e** The lower incision is made from the right to the left anterior axillary lines beneath the breasts (red arrowheads) to create the bipedicle anterior cutaneous flap. The flap is moved upward (blue arrow). Dashed lines indicate the skin incision lines. **f** The new tracheal stoma is created at a point in the midline (red arrowheads). A, innominate artery; Ao, aorta; B, body of sternum; C, clavicle; M, manubrium of sternum; PA, pulmonary artery; R1, first rib; R2, second rib; R3, third rib; T, trachea; V, innominate vein

Under endotracheal general anesthesia, the patient was placed in the thyroid position to produce cervical extension. The transverse incision following the supraclavicular line was constructed by extending laterally from the central tracheal stoma point to the infraclavicular fossae (Fig. 2a). The skin flap was elevated to each cranial and caudal side to expose the upper border of the clavicles and the sternum body. The sternocleidomastoid muscles were detached from the sternum and clavicles bilaterally, and the pectoral muscles were dissected on either side from the mid sternal line to expose the first and second rib cartilages and medial positions of the first and second intercostal muscles (Fig. 2b). The bilateral margins of the sternum in the second intercostal space were exposed, and the internal mammary vessels were dissected bilaterally. Using a bone saw, the manubrium was divided along the midline and transversely at the second intercostal muscle. We then dissected the clavicle heads and first and second rib cartilages (approximately 2 cm from the medial end of the sternum) bilaterally using a bone saw, thereby resolving the bony compression. The trachea was dissected circumferentially to its bifurcation while avoiding damage to the blood supply to the distal end. Anterior mediastinal tissues, such as the thymus, were divided centrally, and the innominate artery and ascending aorta were dissected from the surrounding tissue (Fig. 2c). Trachea was then repositioned to the right side of the aorta and innominate artery and wrapped with thymus to avoid direct touch to the aorta and innominate artery (Fig. 2d). The level of the mediastinal tracheostomy was determined, and the end of the distal trachea was gently moved upward in the mediastinum. To create the bipedicle anterior cutaneous flap, a lower incision was made from the right to the left anterior axillary lines beneath the breasts. The flap was moved upward (Fig. 2e), and the new tracheal stoma was created at a point along its midline (Fig. 2f). The cutaneous defect below the lower incision was covered using a free skin graft from the left thigh. The surgical time was 250 min, with an estimated blood loss of 43 mL.

**Fig. 3:**
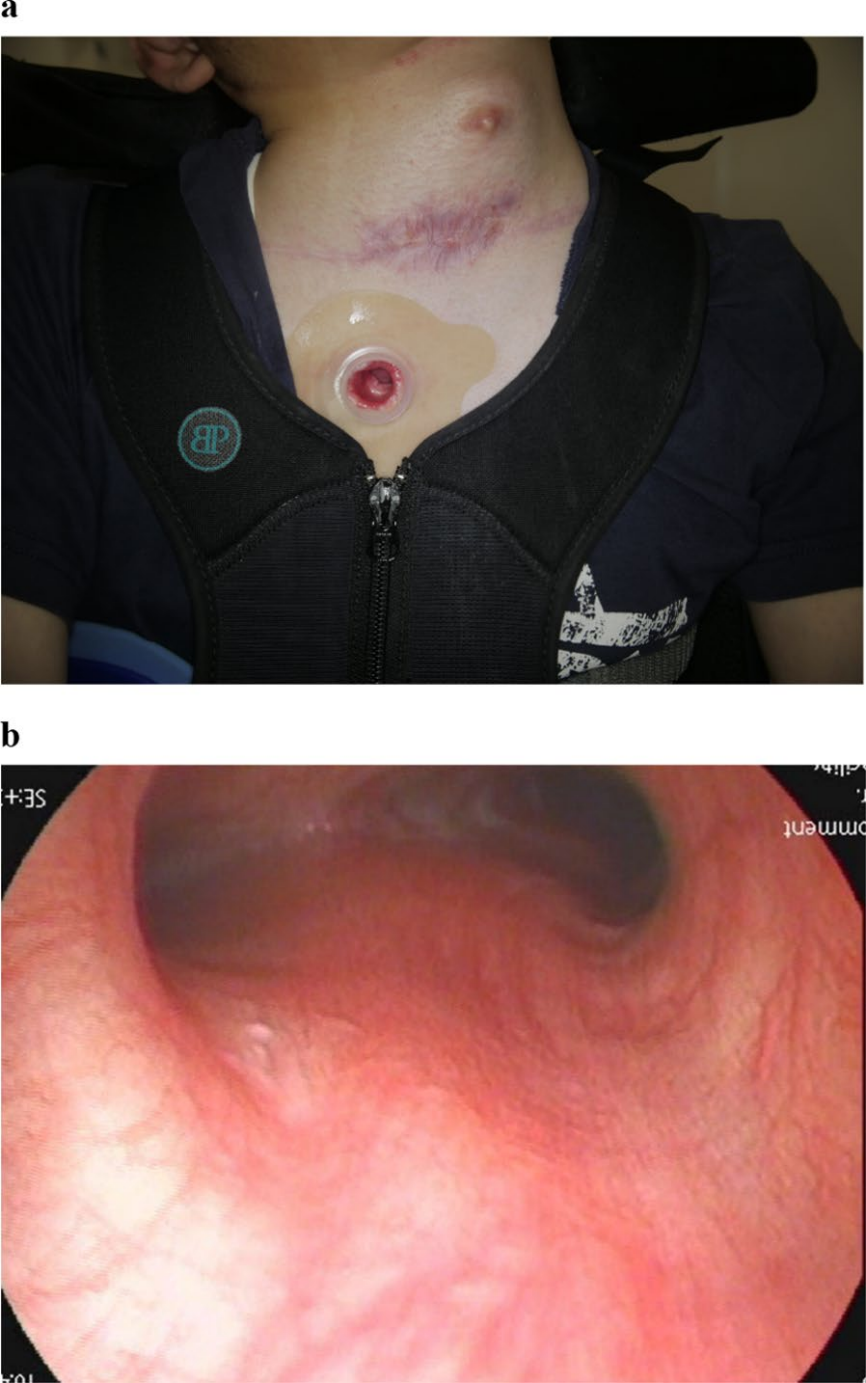
Postoperative findings. **a** The patient is currently living at home without a tracheal cannula. **b** Follow-up bronchoscopy reveals the tracheal stenosis is resolved

Postoperatively, the patient had a supraclavicular wound infection that required only incisional drainage; however, he was discharged home 28 days after surgery without any additional complications. The patient is currently living at home with a 3-year follow-up period after surgery without a tracheal cannula (Fig. 3a), and the tracheal stenosis has been resolved (Fig. 3b).

## Discussion

TIF is known as fatal complication after tracheostomy and laryngotracheal separation. To prevent TIF formation after tracheostomy or laryngotracheal separation, several surgical techniques, including innominate artery transection [10, 11], innominate artery transection with additional revascularization [1, 12], and sternotomy [1, 2, 12] have been reported. However, due to decreased cerebral blood flow or anomalies of the arteries of the circle of Willis, which can occur in up to 54.8% of cases, innominate artery dissection is often contraindicated. Preoperative evaluation by contrast-enhanced CT is necessary in these cases [1, 9, 13]. Revascularization presents additional obstacles, such as artificial vessel infection and the need for graft harvesting. Various methods of sternotomy have been reported [1, 2, 12, 14], but the progressive nature of thoracic deformities and scoliosis in children with SMID requires more extensive resection [14]. Furthermore, sternotomy alone does not resolve the anterior displacement (hyperextension) of the trachea after laryngotracheal separation. We, therefore, opted to perform an anterior mediastinal tracheostomy with repositioned tracheostomy as a comprehensive solution.

Anterior mediastinal tracheostomy, first reported by Grillo, is the standard procedure in cases of head and neck malignancies requiring extensive tracheal resection [15]. This method involves removing the anterior chest wall by resecting the manubrium, clavicles, and ribs, moving the trachea to the right side of the innominate artery, and creating a tracheal stoma in the anterior mediastinum. The advantages of this technique include the following: no need for brachiocephalic artery dissection, sufficient bony compression release, release of the tracheal hyperextension, relief of tracheal and innominate artery contact, and a cannula-free tracheostomy. Conversely, disadvantages include the risk of infection, including wound infection, osteomyelitis, and mediastinal infection, and loss of the sternoclavicular joint. In patients with advanced cancers, extensive mediastinal exenteration, radiation therapy, and chemotherapy can result in dead cavities, decreased blood flow, and tissue fragility, which can exacerbate the risk of infection. However, the background of children with SMID is different from that of malignant cases, and children with SMID have a smaller risk of infection than patients with malignancies. Additionally, the loss of the sternoclavicular joint raises concerns about the possibility of limitation of upper limb mobility. However, in children with SMID, limitation of upper limb motion is unlikely to create additional problems in capacity for activities of daily living. To date, there have been no reports on using this surgical approach to avoid tracheal stenosis or TIF formation in children with SMID. Since this technique is a radical solution to release tracheal compression and hyperextension while preventing TIF, it should be considered in cases with compatible patient background and the skills of the surgical team.

## Conclusions

We report a case of a child with SMID treated with an anterior mediastinal tracheostomy to resolve tracheal stenosis and prevent TIF. Anterior mediastinal tracheostomy could be a good surgical option for cases of severe tracheal stenosis and TIF after tracheostomy or laryngotracheal separation in children with SMID.

## Data Availability

Date sharing is not applicable to this article as no datasets were generated or analyzed during the current study.

## Abbreviations

SMID: Severe motor and intellectual disabilities
TIF: Tracheoinnominate artery fistula
CT: Computed tomography
